# Top-Down NO_X_ Emissions of European Cities Based on the Downwind Plume of Modelled and Space-Borne Tropospheric NO_2_ Columns

**DOI:** 10.3390/s18092893

**Published:** 2018-08-31

**Authors:** Willem W. Verstraeten, Klaas Folkert Boersma, John Douros, Jason E. Williams, Henk Eskes, Fei Liu, Steffen Beirle, Andy Delcloo

**Affiliations:** 1 Royal Meteorological Institute of Belgium (RMI), Ukkel, B-1180 Brussels, Belgium; andy.delcloo@meteo.be; 2 Royal Netherlands Meteorological Institute (KNMI), 3731 GA De Bilt, The Netherlands; boersma@knmi.nl (K.F.B.); john.ntouros@knmi.nl (J.D.); Jason.williams@knmi.nl (J.E.W.); Eskes@knmi.nl (H.E.); 3 Environmental Sciences Group, Wageningen University, 6700AA Wageningen, The Netherlands; 4 Universities Space Research Association (USRA), GESTAR, Columbia, MD 21046, USA; fei.liu@nasa.gov; 5 NASA Goddard Space Flight Center, Greenbelt, MD 20771, USA; 6 Max-Planck-Institut für Chemie, 55128 Mainz, Germany; steffen.beirle@mpic.de

**Keywords:** tropospheric NO_2_ column, surface NO_X_ emissions, OMI data, LOTOS-EUROS CTM

## Abstract

Top-down estimates of surface NO_X_ emissions were derived for 23 European cities based on the downwind plume decay of tropospheric nitrogen dioxide (NO_2_) columns from the LOTOS-EUROS (Long Term Ozone Simulation-European Ozone Simulation) chemistry transport model (CTM) and from Ozone Monitoring Instrument (OMI) satellite retrievals, averaged for the summertime period (April–September) during 2013. Here we show that the top-down NO_X_ emissions derived from LOTOS-EUROS for European urban areas agree well with the bottom-up NO_X_ emissions from the MACC-III inventory data (R^2^ = 0.88) driving the CTM demonstrating the potential of this method. OMI top-down NO_X_ emissions over the 23 European cities are generally lower compared with the MACC-III emissions and their correlation is slightly lower (R^2^ = 0.79). The uncertainty on the derived NO_2_ lifetimes and NO_X_ emissions are on average ~55% for OMI and ~63% for LOTOS-EUROS data. The downwind NO_2_ plume method applied on both LOTOS-EUROS and OMI tropospheric NO_2_ columns allows to estimate NO_X_ emissions from urban areas, demonstrating that this is a useful method for real-time updates of urban NO_X_ emissions with reasonable accuracy.

## 1. Background

High levels of nitrogen oxides (NO_X_ = NO + NO_2_) are toxic and adversely impact both human health [1] and ecosystems [2,3]. NO_X_ is mainly generated in polluted regions by anthropogenic combustion of fuels from traffic, industrial processes and household activities that typically occur in densely populated urban areas. In 2013 for the EU-28, 46% of the anthropogenic NO_X_ emissions originated from the transport sector, 21% from the energy and 15% from the industry sector [4], excluding emissions from international shipping within European seas. According to the European Environment Agency, around 10% of the urban population in the EU-28 is exposed to air pollutant concentrations above EU and WHO reference levels (2011–2013) for NO_2_ [4]. Since 86% of the exceedances are measured at traffic stations, the percentage of the urban population exposed to high NO_2_ concentrations may be somewhat underestimated as >20% of the European urban population now lives less than 400 m from busy roads [5]. Hence, millions of people may potentially suffer from health issues and loss of productive labour. 

NO_X_ controls the photochemical formation rate of O_3_ and thus the production of the hydroxyl radical and hence the chemical lifetime of key atmospheric pollutants and reactive greenhouse gases [6,7]. An end product of the NO_X_ reaction chain is nitric acid, which acts as an efficient aerosol pre-cursor and an acidifying substance. Accurate NO_X_ emission inventories are essential in order for regional and global chemistry transport models (CTM) to capture the lifetimes and mixing ratios of tropospheric pollutants. However, current state-of the-art emission databases vary substantially and uncertainties are high [8,9,10] since reported emissions factors may differ by an order of magnitude or more. For example, NO_X_ emissions of diesel cars have been underestimated up to 20-fold in officially announced data [11]. Moreover, the most recent emission estimates proposed for the latest round of the Climate Model Intercomparison Project result in 10–15% more annual NO_X_ emissions than previous estimates [12]. 

Satellite sensors have proven records in the retrieval of tropospheric NO_2_ columns over many large cities worldwide [10,13,14,15,16]. From these NO_2_ observations NO_2_ lifetimes and up-to-date top-down NO_X_ emissions can be derived [10,15,16,17,18,19,20,21,22,23] demonstrating the ability of satellite data to bridge the gap between actual emission fluxes and the reported inventory data, which typically have a delay of 3–5 years before they are available.

## 2. Objectives

Modelled tropospheric NO_2_ concentrations and lifetimes have large uncertainties associated with them [24] due to the highly non-linear small-scale chemistry that occurs in urban areas, uncertainties in the chemical reaction rate data, missing nitrogen (N) species, too low volatile organic compounds (VOC) emissions, incomplete knowledge of NO_X_ chemistry reaction coefficients, extended chemical reactions [7,25] and recycling of NO_X_ via nitrate photolysis [26]. Overestimation in the chemical lifetime of NO_2_ may point out missing NO_X_ chemistry in current CTM’s [25]. By simultaneously estimating both the NO_2_ lifetime and NO_2_ emissions from the downwind plume evolution independent NO_X_ emission flux estimate can be derived [10]. 

A frequently applied and proven methodology to estimate the lifetime of NO_X_ from satellite observations is based on the downwind plume of tropospheric NO_2_ data that evaluates the effect of pre-selected wind speed and wind direction on observed tropospheric NO_2_ distributions over and downwind of pollution hotspots. This method is not considering vertical mixing and vertical profiles of NO_X_/NO_2_ ratios but is almost free of a-priori assumptions or model input [10,15,16]. 

An important goal of this study is to test the downwind plume method for European cities that so far has only been applied directly on observed tropospheric NO_2_ column fields for US and Chinese cities and power plants. We also evaluate to what extend the known a-priori emissions used in a high-resolution model simulation of tropospheric column fields over European cities are represented using the adapted method for polluted backgrounds of Liu et al. (2016) [16]. Instead of assuming point sources, this method uses the mean NO_2_ distribution under calm conditions as proxy for the spatial distribution of NO_X_ emissions and derives the NO_2_ lifetime from the difference of NO_2_ patterns between calm and windy conditions.

Many European cities cannot be considered as point sources of NO_X_ given the short distances between cities and other sources close to cities. For example, the distance between Brussels and Antwerp with a major port is less than 50 km. So, pollution of these cities overlaps and is smeared out from multiple sources over a larger area. 

The magnitude of the discrepancies between the bottom-up NO_X_ emissions and the top-down estimates derived with the downwind plume approach provide a direct test of the robustness of the method and can be used to quantify the uncertainties in the NO_X_ emissions and lifetime estimates. We show that a small dataset of only one season (April–September 2013) can be used to derive robust values of NO_X_ emissions and NO_2_ lifetimes.

Tropospheric NO_2_ column model fields simulated with the LOTOS-EUROS (Long Term Ozone Simulation-European Ozone Simulation, [27]) CTM averaged for the period April–September 2013 over 23 selected European urban areas under windy conditions (averaged surface to 500 m wind speeds >5 m s^−1^) were used in the downwind plume approach adapted for polluted backgrounds. As an end-to-end test, we then compare the top-down derived surface NO_X_ emissions with the 2011 MACC-III emission inventory [28], used in the LOTOS-EUROS model as input to simulate the NO_2_ columns. We compare the NO_2_ lifetimes derived from LOTOS-EUROS with values reported in the literature and we discuss possible cross-correlations between the lifetime and emission estimates. 

After demonstrating that the downwind plume method works on model fields and estimating the intrinsic uncertainties of the method, we apply it on OMI (Ozone Monitoring Instrument) tropospheric NO_2_ column data, providing us with real-time observation-based estimates of midday NO_2_ lifetime and NO_X_ emissions over 23 European cities in 2013. Where available in these cities, the top-down estimated NO_X_ emissions are also compared with reported surface NO_2_ concentrations which are converted to NO_X_ emissions using the LOTOS-EUROS CTM. In Section 3 the used datasets are described. Both the OMI tropospheric NO_2_ column retrieval and processing (Section 3.1) as well as the LOTOS-EUROS tropospheric NO_2_ data (Section 3.2) are discussed. We also make use of surface NO_2_ concentrations over some European cities (Section 3.3). In Section 4 we describe the methodology of the downwind plume approach applied on European cities in polluted backgrounds. In Section 5 we present the results and add discussions on the NO_2_ lifetime (Section 5.1), NO_X_ the emissions (Section 5.2) and the involved uncertainties (Section 5.3). In Section 6 we wrap-up with a summary and conclusions.

## 3. Datasets

### 3.1. OMI Tropospheric NO_2_ Column Retrieval and Processing

The 2013 April to September OMI tropospheric NO_2_ columns are retrieved (DOMINO v2 [29]) with a three-step approach: (i) a retrieval of NO_2_ slant columns with Differential Optical Absorption Spectroscopy (405–465 nm wavelength range, spectral resolution of 0.5 nm), (ii) assimilation of OMI NO_2_ slant columns in the global CTM TM4 for providing stratospheric background columns and a-priori NO_2_ profile shapes needed to calculate the tropospheric air mass factors [30], which are then used to (iii) convert the tropospheric slant columns into vertical columns. The OMI pixel size varies from 24 km × 13 km at nadir to 140 km × 26 km at the edges of the swath. Individual OMI pixels were re-gridded to 0.125° latitude by 0.125° longitude. Pixels affected by the OMI row anomaly were excluded from the analysis [29]. It has been shown that OMI tropospheric NO_2_ vertical column densities exhibit substantial sensitivity to boundary layer NO_2_ levels [29,30,31,32] and the DOMINO v2 product has been used and validated extensively [33]. Retrievals with cloud fractions higher than 30% at ~13h30 local time are not considered in the analysis.

### 3.2. LOTOS-EUROS Datasets

LOTOS-EUROS is a 3D CTM with focus on simulating the atmospheric composition in the lower troposphere [27]. For this study, the model was driven by ERA-Interim meteorological fields from the ECMWF (European Centre for Medium-Range Weather Forecasts) and the emission inventory developed by TNO (Netherlands Organisation for Applied Scientific Research) for the MACC-III project (Monitoring Atmospheric Composition & Climate) and covers Europe with a resolution of 7 × 7 km^2^ [28]. Model simulations were performed for Europe (25° W–45° E, 30° N–70° N) with a spatial resolution of 0.25° by 0.125° with MACC-III emissions data updated until 2011 as input. In the vertical domain, the model uses a bulk boundary layer scheme with four layers. The first layer is the surface layer of 25 m and layer 2 is a single boundary layer with a thickness depending on the time of day. The layer 2 height is obtained by temporal interpolating the boundary layer height field provided by ECMWF, available every three hour. Layers 3 and 4 are reservoir layers and the top of the model is set to 3.5 km height. The NO_2_ dataset used in this study was produced using version 1.10 of the model, which was run in forecast mode. The tropospheric NO_2_ column datasets derived from the model are columns collocated at the OMI measurement points using LOTOS-EUROS data up to the model top (3.5 km) and then complemented by MOZART-IFS data [34] for the rest of the troposphere (defined at 200 hPa).

### 3.3. Surface NO_2_ Measurements

Surface NO_2_ measurements of European cities are available from the website http://www.eea.europa.eu (Air Base). Since monthly anthropogenic NO_X_ fluxes do not change significantly throughout the year, an average retrieved value will provide a good estimate [27,35]. Measured surface NO_2_ concentrations were converted to NO_X_ surface emission values by assuming that the observed NO_X_/NO_2_ ratio at the surface approximates the modelled NO_X_/NO_2_ ratio obtained from the MACC-III NO_X_ emissions and the corresponding LE simulated NO_2_ concentrations for the model surface layer. This scaling approach can be written as:

E_NO_X___,obs_ = (E_NO_X___,LE_/NO_2,LE_) × NO_2,obs_ (1)

where E_NO_X___,LE_ is the MACC-III input NO_X_ emission, NO_2,LE_ the concentration of NO_2_ at the surface layer in LE, NO_2,obs_ the observed NO_2_ surface concentration and finally E_NO_X___,obs_ the scaled NO_X_ emission. Next numerical example for Brussels is given as illustration. In Brussels, an annual mean NO_2_ of 33.44 µg m^−3^ is observed, corresponding to 13.80 ppb. The LOTOS-EUROS model simulates a NO_2_ concentration at the surface layer of 17.20 ppb for a surface NO_X_ emission of 4.98 mg m^−2^ day^−2^ over the grid cell of Brussels. The estimated surface NO_X_ emission based on the observed NO_2_ concentration is then estimated as 13.80 ppb/17.20 ppb × 4.98 mg m^−2^ day^−1^ = 3.99 mg m^−2^ day^−1^.

## 4. Methods

**Figure 1:**
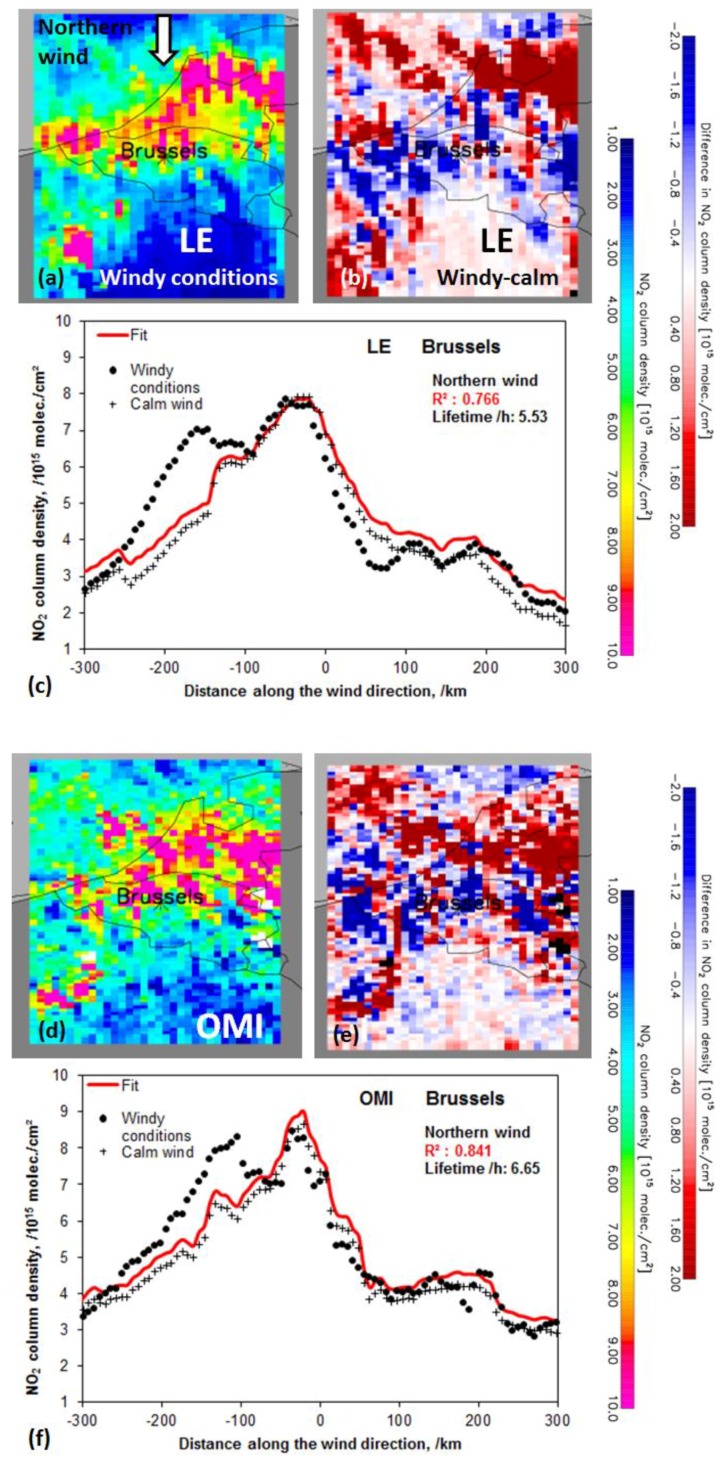
Spatially explicit LOTOS-EUROS (Long Term Ozone Simulation-European Ozone Simulation) (**a**) and Ozone Monitoring Instrument (OMI) (**d**) tropospheric NO_2_ columns for winds coming from the north centred around Brussels according to the major up & down-wind distance of ~300 km and an across wind direction distance of ~100 km sampled on available OMI data on a 0.125° × 0.125° grid for windy conditions (>5 m s^−1^) averaged over the period April–September 2013. The difference map between windy and calm conditions for LOTOS-EUROS (**b**) and OMI (**e**). The averaged tropospheric NO_2_ columns of Brussels converted to upwind and downwind line densities LOTOS-EUROS (**c**) and OMI data (**f**) for both calm as windy conditions. The fit of the exponential function under windy conditions and the observed NO_2_ pattern under calm conditions are convolved using Equations (2a) and (2b) and is given in red. The corresponding effective lifetime and R^2^ values of the fit are also shown.

In order to test the downwind plume approach on model fields, we sampled LOTOS-EUROS (LE) tropospheric NO_2_ columns at locations where OMI cloud fractions (determined from the OMI O_2_-O_2_ retrieval [36]) are below 30% at ~13h30 local time to ensure that both OMI and LOTOS-EUROS are sampled for comparable clear-sky circumstances. We then re-grid both datasets onto a common 0.125° × 0.125° grid, so that both datasets are directly comparable. Next, we compute for each grid cell the prevailing wind direction (sector) from eight possible choices (N, NE, E, SE, S, SW, W and NW) using ECMWF ERA-interim wind direction data [37]. For each wind sector, we select two datasets of tropospheric NO_2_ columns. One dataset for calm conditions and one dataset for windy days using the wind speed threshold of <5 m s^−1^ (averaged from surface up to 500 m altitude) [10,15,16] in order to have sufficient spatial coverage for both wind conditions. Finally, we averaged the data over the period April–September 2013. For every eight wind sectors, we then compute a subset of tropospheric NO_2_ for a chosen European city according to the major up and down-wind distance of ~300 km (~600 km in total) and an across wind direction distance of ~100 km (~200 km in total) for both OMI and LE. This procedure is illustrated in Figure 1 for respectively the LE (Figure 1a) and OMI tropospheric NO_2_ columns (Figure 1b) for the city of Brussels for the case of winds coming from the north. Wind sectors with gaps due to missing data larger than 10% in the across-wind integration interval and larger than 20% in the fit interval in wind direction are not used for the retrievals of lifetimes and NO_X_ emissions [16]. The panels (a) and (d) in Figure 1 show the complex patterns of the NO_2_ wind plumes due to the many local NO_X_ sources with high tropospheric NO_2_ columns in the middle and south of the Netherlands as well as in Flanders (North-Belgium) and the relatively lower values south of Brussels in Wallonia. From the difference maps of NO_2_ columns between windy and calm conditions in panels (b) and (e) of Figure 1 the relatively higher NO_2_ values up-wind and relatively lower NO_2_ values down-wind the Brussel city centre can be observed considering the applied large across wind interval of 200 km. 

Next, we have computed for each wind sector the NO_2_ line density function over a distance of ~300 km downwind and ~300 km upwind from the city centre [15,16]. By averaging the NO_2_ data over the specified across-wind interval (2 × 100 km), the NO_2_ line densities are obtained as illustrated in Figure 1 for Brussels using LE (panel (c)) and OMI tropospheric NO_2_ columns (panel (f)) for each wind sector for both calm (indicated by +) as windy conditions (>5 m s^−1^) (indicated by ●).

The first tropospheric NO_2_ peak of ~7 × 10^15^ molec. cm^−2^ in the NO_2_ line density for the LE case (panel (c) of Figure 1) is observed at ~150 km north of Brussels and originates from the high tropospheric NO_2_ values over the mid-Netherlands ranging from west to east. The second tropospheric NO_2_ peak (~8 × 10^15^ molec. cm^−2^) occurs just north of Brussels and is the result of averaging the accumulated high NO_2_ columns from west to east from the Flemish coast over parts of Rotterdam, Antwerp and further to the east. South from Brussels, the line density drops rapidly due to the more rural character of the area. The little tropospheric NO_2_ peaks (~4 × 10^15^ molec. cm^−2^) at 100 and 200 km south of Brussels are due to the inclusion of NO_2_ hotspots over Saint-Quentin and Paris, respectively. South of Paris the tropospheric NO_2_ values drop to background values.

In order to derive the NO_2_ lifetime and NO_X_ emissions from the line densities of tropospheric NO_2_ columns, we follow the approach of Reference [16] for polluted backgrounds. The NO_2_ spatial pattern under calm wind conditions (polluted background field) are used as proxy for the distribution of NO_X_ sources, that is, multiple sources of NO_X_ are accounted for (as observed on the maps in f panels (a) and (d) of Figure 1). The effective atmospheric NO_2_ lifetime is determined from the change of spatial patterns measured at windy conditions. Emissions are subsequently derived from the NO_2_ mass above the background, integrated around the source of interest. Next convolution function of tropospheric NO_2_ under calm conditions with the exponential decay function under windy conditions is fit on the data:

NO_2,line_(x|x_0_, µ, α, β) = α · [e⊗C](x) + β, (2a)

e(x) = exp(−(x − µ)/x_0_) for x ≥ µ, 0 otherwise, (2b)

C(x) is the NO_2_ pattern under calm wind conditions; x_0_ is the length scale of the NO_2_ decay; µ is the location of the apparent source relative to the city centre; α and β are scaling parameters to account for possible differences between windy and calm wind conditions (e.g., cloud conditions, vertical profiles, lifetimes [16]). The accompanying fits of the convolved function using NO_2_ observations for calm wind condition and the exponential decay function on the windy condition data of Equation (2) for each wind direction are shown in Figure 1. This procedure was then applied on 23 selected European urban areas as presented in Table 1. The effective lifetime is then derived by the ratio of the fitted e-folding distance (x_0_) (decay time) and the mean ECMWF derived wind speed (ω) like in Reference [10] (Equation (3)) but we have subtracted the residual mean wind speed under calm wind conditions from the windy conditions as suggested by [16] indicated as ω*:

τ_effective_ = x_0_/ω*, (3)

The assumption is that the removal of NO_2_ can be described by a first order loss. Stated otherwise, the chemical decay of NO_2_ follows an exponential decay function with an e-folding distance x_0_, which results in an overall and effective NO_2_ lifetime. For Brussels, the effective lifetime based on OMI observations is 6.7 h for the N wind direction with a fit R^2^ value of 0.84 (see also Figure 1, panels (c) and (f)). Similar to Reference [16], the fit results are averaged for wind sectors with a good fit of the convolution function (R^2^ > 0.80 and lower bound >0 h and <10 h) (in this case 4.5, 5.1, 6.7, 3.2 h for SW, W, N, NE respectively) and weighted by the R^2^ values (0.92, 0.81, 0.84, 0.92, respectively) which yields 4.8 ± 2.3 h. The effective lifetime based on LE data weighted for the different wind sectors is 3.8 ± 2.5 h (see also Figure 1), which is slightly lower than the derived lifetime for the OMI case. 

**Figure 2:**
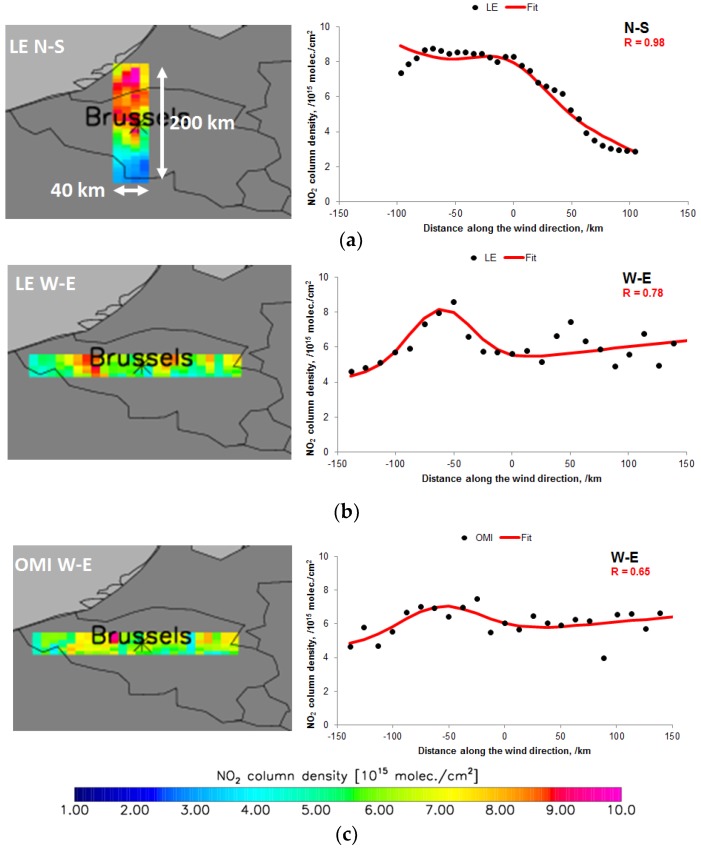
The averaged tropospheric NO_2_ columns of Brussels for the period April–September 2013 for calm wind conditions for LOTOS-EUROS data (LE) and OMI data. The maps on the left show the NO_2_ patterns for their specific wind directions using a 200 km range along and 40 km across the wind direction as indicated. The graphs on the right show the corresponding line densities with Equation (4) fit on the data. (**a**) shows the LE NO_2_ patterns and line densities for N-S winds, (**b**) shows the LE NO_2_ patterns and line densities for W-E winds, (**c**) shows the OMI NO_2_ patterns and line densities is for N-S winds.

Based on the mass balance, the total mass of NO_X_ is the product of the emission rate with lifetime. We follow the methodology described by Reference [16] to estimate the emissions using a three-step approach by computing the total mass of NO_2_ (i), then scale NO_2_ to NO_X_ (ii) and (iii) finally dividing the total NO_X_ mass by the lifetime. More detailed, at first (i) we integrate the observed NO_2_ columns over the area source of interest to compute the total mass of NO_2_ while avoiding interferences with neighbouring sources and by accounting for the polluted background using line density functions under calm wind conditions computed for a smaller interval across (40 km) and along (200 km) wind directions. A nonlinear least-squares fit of a modified Gaussian function g(x) (see Equation (4)) to the NO_2_ line densities under calm wind condition for the different wind sector pairs (N-S, E-W, NE-SW and NW-SE) is performed. Figure 2 shows this procedure for Brussels.

g(x) = A·(2πσ)^−1/2^·exp(−(x − μ)/(2σ^2^) ) + ε + β · x, (4)

For each wind sector pair, the total amount of NO_2_ molecules is given by A, the relative source centre is µ, the standard deviation of the Gaussian function is σ, β is the slope of the linear part of the function and ε is the offset (background). By fitting the functions g(x) simultaneously for all available wind sector pairs, the total amount of NO_2_ molecules (parameter A) around the source is determined. Only the best fits of the g(x) functions are used. The fit of total NO_2_ mass is performed over an interval in the wind direction which is set to 200 km (see Figure 2) accordingly to Reference [16] in order to have a meaningful fit of g(x). This interval potentially includes interfering NO_X_ sources but is indirectly accounted for by the linear variation of the background fit. The small across-wind interval (40 km) (see Figure 2) excludes neighbouring sources but does not capture the full plume in across wind direction due to dilution which is corrected by scaling A afterwards by a factor based on the ratio of the Gaussian function integrated over −20 and +20 km and the Gaussian function integrated between −∞ and +∞.

Next, (ii) in order to derive total NO_X_ mass, NO_2_ to NO_X_ is scaled by computing local scaling factors from LOTOS-EUROS, which typically range from 1.31 to 1.56 at 13h30 local time. This scaling is compared with the factor of 1.32 as applied in Reference [10]. 

Finally, (iii) the NO_X_ emission rates (mg m^−2^ day^−1^) are derived by dividing of the total NO_X_ mass by the lifetime. We use milligrams of NO_X_ per square meter per day in order not to make any assumptions on the exact area of each individual city. For Brussels, based on OMI observations, the total NO_X_ mass per unit area is 741.6 mg m^−2^, with the NO_2_:NO_X_ scaling of 1.43 instead of 1.32. Using the derived effective lifetime of 4.8 h (per day 4.8 × 24), the total NO_X_ emissions derived for Brussels is 6.42 mg m^−2^ day^−1^. Based on the LE tropospheric NO_2_ data, the total NO_X_ emission is 4.90 mg m^−2^ day^−1^. The top-down derived NO_X_ emissions are also compared with airbase surface NO_2_ concentrations converted to emissions based on the LE data as described in Section 3.3.

## 5. Results and Discussions

### 5.1. Lifetimes

Table 1 summarizes the results of the downwind plume method applied to the LOTOS-EUROS and OMI averaged April-September 2013 tropospheric NO_2_ column distributions over 23 European cities. On average, for the cities the effective NO_2_ lifetime is 4.1 ± 2.2 h derived from OMI tropospheric NO_2_ columns and 3.7 ± 2.0 derived from LE simulated tropospheric NO_2_ columns. The slight overall difference in NO_2_ lifetimes is small and not significant (*t*-test, *p*-value > 0.34). For some cities (Amsterdam, Warsaw, Paris, Milan, Marseille, Rome, Naples, Istanbul), the OMI derived lifetimes are smaller than the LE derived values but in general the differences are small. Only for Rome and Istanbul the discrepancies are larger (1.8 vs. 3.5 h and 4.5 vs. 6.4 h respectively) (Table 1). 

For Rome, the lifetime for LE is derived for a wind speed threshold of 3 m s^−1^, for western winds since no reasonable fit was found at the threshold of 5 m s^−1^. The OMI derived lifetime is for southwestern winds at speeds of >5 m s^−1^. Reference [19] reports that for higher wind speeds generally shorter lifetimes are observed. For Istanbul, the OMI peak NO_2_ column (>4 × 10^15^ molec. cm^−2^) is much larger and contrasts more with the background (~2 × 10^15^ molec. cm^−2^) than for the LE data (peak at ~2 × 10^15^ molec. cm^−2^ and background of ~1 × 10^15^ molec. cm^−2^). Thus, the LE NO_2_ plume decays much faster suggesting a shorter NO_2_ lifetime. For London, Antwerp, Brussels, Kiev and Vienna, the LE derived lifetimes tend to be lower than the OMI ones. This might be due to the fact that OMI NO_2_ is more smeared and smoothed with higher background NO_2_ concentrations (see Figure 1) while LE NO_2_ values show large differences between hotspots and background NO_2_ concentrations (see also in Figure 1), which is in agreement with literature reports [38]. Again, these substantial differences between OMI and LE background NO_2_ concentrations (see also in Figure 1) may slow-down the decrease in NO_2_ columns away from the city centre, leading to a higher NO_2_ lifetime. 

The average lifetimes (4.1 h for OMI and 3.7 h for LE, see Table 1) are similar to the reported ones in the literature as shown in Table 2. Our LE NO_2_ effective lifetimes of Madrid and Moscow (4.6 and 4.1 h respectively, Table 1) are in the same order of magnitude than the estimates from [10] with slightly different parameters settings (the across-wind direction was set to 100 km for eight wind sectors and wind speeds >2 m s^−1^). The OMI derived lifetimes for these cities are slightly higher than the LE based estimates (5.7 and 5.4 h, respectively), given the higher contrast between peak and background NO_2_ in LE. For Helsinki and St-Petersburg the LE-based NO_2_ lifetime estimates (3.1 and 3.8 h, respectively) are similar to the reported values by [14] but the OMI derived lifetimes are slightly higher (4.7 and 3.9 h, respectively). 

This could be due to different parameter settings, especially for the across-wind intervals used wind sectors. Summer season NO_2_ [10,16] tends to lower lifetimes compared to spring and autumn due to higher OH concentrations promoting conversion to HNO_3_. As in Reference [16], no overall correlation exists between NO_2_ lifetimes and tropospheric NO_2_ columns, NO_X_ emissions, wind speed or latitude (all R^2^ < 0.03). This is probably due to the complex NO_X_ chemistry which is also affected by meteorological and chemical variability, like variations in UV flux, water vapour and VOC levels [16].

### 5.2. NO_X_ Emissions

**Figure 3:**
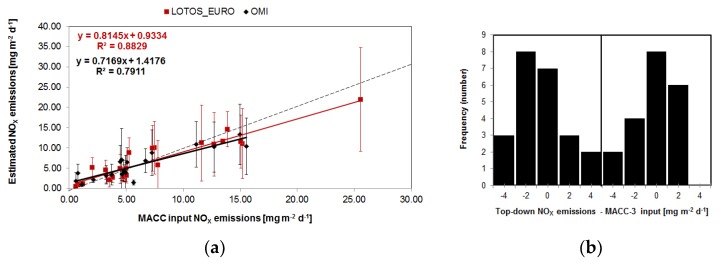
Top-down derived NO_X_ emissions estimated from the tropospheric NO_2_ line densities for different European cities against the MACC-III emissions as used in LOTOS-EUROS (**a**). NO_X_ emissions from LOTOS-EUROS versus MACC-III in red. OMI based versus MACC-III in black. For each NO_2_ data set the statistics (slope, intercept, correlation coefficient) are provided. The OMI derived emission of 4.28 ± 1.68 mg m^−2^ day^−1^ for St.-Petersburg is omitted from the figure. (**b**) The distribution of the difference of the top-down and MACC-3 input emissions.

For all the selected European cities, the averaged top-down NO_X_ emissions derived from LE (7.11 ± 4.03) are similar to the MACC emissions input into the LE model (7.59 ± 3.97 g m^−2^ d^−1^) and certainly within reported errors [10,15,16]. This demonstrates the ability of the applied methodology on the selected cities to reproduce NO_X_ emissions based on tropospheric NO_2_ columns derived from a short time period. Figure 3 shows the top-down derived NO_X_ emissions estimated from the tropospheric NO_2_ line densities for different European cities against the MACC emissions as used in LOTOS-EUROS (see Table 1) and the corresponding slopes, intercepts and R^2^ values. A strong correlation exists for both the OMI (without St.-Petersburg as explained below) as LE derived emissions against the model input (R^2^ = 0.79 and 0.88 respectively). Panel (b) of Figure 3 shows the distribution of the difference between the top-down and MACC-3 input emissions for both LE (negatively skewed) as well as the OMI data (slightly positively skewed). Simple *t*-tests between the OMI-MACC and LE-MACC data pairs show that the differences are not significantly different from zero (OMI: *p*-value > 0.32; LE: *p*-value > 0.79). These R^2^ values are similar to the reported values (R^2^ = 0.87 for the US, [15]; R^2^ = 0.74–0.87 for China and the US, [16]). Since the estimated emissions are directly influenced by the [NO]:[NO_2_] ratio, we substituted the general factor 1.32 [10] with the one directly computed from the modelled [NO]/[NO_2_] concentration ratio for each city. The LE based ratios range between 1.31 and 1.56. Using the generic 1.32 factor (which is in general only 5% lower than the variable scale factors), the correlation between the MACC input and the top-down emissions remains high (R^2^ = 0.77 and 0.88 for OMI and LE respectively) but the NO_X_ emission values are 6.5 and 8.3% lower. 

For St-Petersburg the OMI derived top-down emission estimates is 4.28 ± 1.68 mg m^−2^ day^−1^ which is notably lower than the corresponding MACC value (27.02 ± 26.55 mg m^−2^ day^−1^) while the LE derived emissions are comparable (21.95 ± 12.79). This might be due to rapid changes in anthropogenic activities [39] that are not captured by the MACC inventory. A decline in the NO_X_ emission trend for the period 2011–2014 over large parts of Europe was reported [23], which might explain the lower OMI top-down estimates for 2013 compared with MACC-III (2011 update) in our study for most of the selected cities. The average peak tropospheric NO_2_ column (maximum value in the line density functions) for St-Petersburg from LE is ~12 × 10^15^ molec. cm^−2^, while OMI only observes ~8 × 10^15^ molec. cm^−2^ in 2013 (and ~6 × 10^15^ molec. cm^−2^ in 2012). The lower derived emissions from OMI may reflect the slow-down in the Russian economic growth (1.3% in 2013 versus 3.4% in 2012 and only ¼ of the growth during the last decade, [40]) and by the slightly decrease in anthropogenic emissions (0.8% decrease in CO_2_ from cement production, less CO_2_ emissions per energy production unit and per capita, 1.5% reduction in NO_X_ emissions) [40] and is consistent with reported reductions of tropospheric NO_2_ by [38]. Interesting and not completely clear is that for the city of Moscow both OMI as well as the LE top-down NO_X_ estimates correspond well with the MACC emissions. The OMI NO_2_ peak is ~6 × 10^15^ molec. cm^−2^ while the LE peak is 8 × 10^15^ molec. cm^−2^. Although this is 33% higher, this might be offset by the 30% higher lifetime for OMI (5.4 h vs. 4.1 h). 

**Figure 4:**
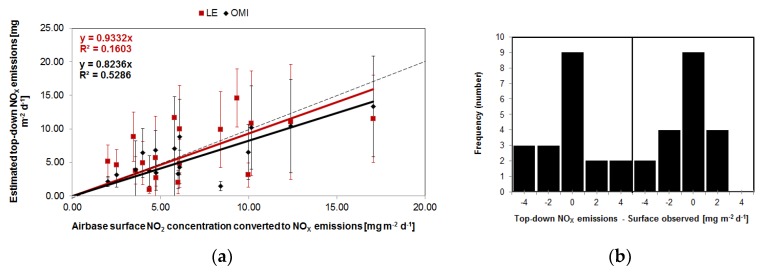
(**a**) Top-down derived NO_X_ emissions estimated from the tropospheric NO_2_ line densities for different European cities against scaled Airbase measurements of surface NO_2_ concentrations. NO_X_ emissions from LOTOS-EUROS versus MACC-III in red. OMI based versus MACC-III in black. No surface data available for St.-Petersburg, Moscow and Kiev. (**b**) The distribution of the difference of the top-down and surface based observed emissions.

Scaled Airbase measurements of surface NO_2_ concentrations to surface emissions using the LE model are 13% higher than the top-down derived NO_X_ emissions estimated from OMI tropospheric NO_2_ line densities and 11% lower than the LE derived values. However, the correlation (R^2^) between these scaled values and OMI derived emissions is 0.53 and much higher than for the LE derived emissions (R^2^ = 0.16) (Figure 4). This might be attributable to the fact that both OMI as the Airbase data are real-time observations, rather than modelled data without the timely updated emissions input. For cities such as London, Prague, Budapest and Madrid the surface data derived emissions are substantially larger than the OMI derived ones (1.2 to 5.8 times). This might be due to surface measurements being conducted in hotspot emission areas which correspond to high observations that cannot be captured by the coarser spatial resolution data used in the top-down methodology (values are more smoothed and area averaged). In general, background surface measurements agree better with the MACC emissions compared with the surface measurements including urban traffic data. The difference between the top-down and converted emissions based on surface NO_2_ observations are centred around zero (panel (b) in Figure 4).

### 5.3. Uncertainties

The downwind plume methodology is prone to different sources of error. Some errors only affect the lifetimes or the emissions, some of them affect both. A detailed uncertainty analysis of the approach can be found in Reference [16] and their supplementary information. Summarized, the along and across line density intervals, fit results and wind fields contribute to the uncertainty of both the lifetimes as well as the emissions. 

Changing the along and across intervals with 100 km affects the resulting lifetimes by only about 10%. Fit errors are typically in the order of 30% for the lifetimes and 20% for the emission parameter (A in Equation (3)). The uncertainties associated with the wind data are estimated as 30% and uncertainties due to the fit of the total NO_2_ mass as 20% [16]. The uncertainty in tropospheric NO_2_ column observations from OMI is about 30% [29]. The uncertainty of the NO_X_/NO_2_ scaling factor is estimated to be 10% [10] which is in agreement with the individual scaling factors derived in this study which differ in general only 5% from the 1.32 factor. Last both uncertainties affect the emission estimates only, since lifetimes are derived from the relative decay pattern only. According to Reference [16], the interference between the NO_X_ sources cannot be distinguished within 20 km distance and from 40 km on, the interfering sources will not be included in the emission estimates. 

Following the theory on general error propagation and assuming that all errors are independent, the average error on OMI derived lifetimes is 55% (range 41–81%) based on wind field errors of 30% using sum of squares of the relative errors. The average fitting error is 47% (15–113%), which is much larger than 30% as reported in Reference [16]. The corresponding LE derived lifetime errors are 63% (40–116%) and 53% (16–124%). Adding 30% error to the derivation of the total amount of NO_2_ molecules (parameter A in Equation (3)), plus 10% on the 1.32 scaling factor and 30% on the OMI NO_2_ retrieval or LE NO_2_ simulation as done in Reference [10], we can estimate the error on the computed NO_X_ emissions. Based on the OMI data, we have on average 55% error on the NO_X_ emissions (41–81%). Using the LE data, an average error of 62% is obtained (40–101%). Reference [10] reports a total error on lifetimes estimates of 36 to 58% and 46–63% for emissions with the highest value of 78%. Reference [16] has estimated that total uncertainties of NO_X_ lifetime and emissions are within 39–80% and 55–91% range, respectively. Compared to [10] and [16] our estimated uncertainty is larger but in the same order of magnitude. The larger uncertainty might be due to the much shorter dataset that we used to estimate lifetimes and emissions only covering one season (April–September 2013) only, while Reference [15] covers 2005–2014 (April–September) and Reference [16] covers the 2005–2013 (May–September) period. Reference [10] used 2005–2009. Despite the results obtained by Reference [41], we show that it is possible to derive the NO_2_ lifetime and NO_X_ emissions using the downwind plume method on a short time period of one season with reasonable accuracy.

## 6. Summary and Conclusions

In order to test the appropriateness of the downwind plume approach to derive surface NO_X_ emissions from tropospheric NO_2_ columns, we applied this methodology on 2013 LOTOS-EUROS (LE) tropospheric NO_2_ model fields for 23 selected European cities and compared the top-down derived NO_X_ estimates with the MACC input NO_X_ model emissions. In addition, the top-down LE derived surface NO_X_ emissions were evaluated with up-to-date NO_X_ estimates obtained by applying the same approach on OMI data and where available we compared it with surface data of NO_2_ concentrations.

Overall, no substantial differences are observed in NO_2_ lifetimes for 23 selected European cities derived from LE and OMI tropospheric NO_2_ columns. In general, the top-down derived NO_X_ emissions from LE and OMI tropospheric NO_2_ column data are comparable with the MACC inventory. Furthermore, in agreement with literature reports, the top-down NO_X_ estimates are biased low with respect to reported surface NO_2_ observations. The total errors on the derived NO_2_ lifetimes and NO_X_ emissions are larger but in the same order of magnitude with reported literature values.

In summary, the downwind plume approach applied to LE tropospheric NO_2_ columns reproduce the MACC NO_X_ emission LOTOS-EUROS model input well, given the applied chemistry schemes and reactions in LOTOS-EUROS. This demonstrates the ability of this technique to estimate up-to-date NO_X_ emissions from other datasets including satellite observations of tropospheric NO_2_ over a short period which can bridge the gap between fixed date inventory emissions and today’s emitted NO_X_ at the surface of many European cities.

## Figures and Tables

**Table 1:** The top-down derived NO_2_ lifetime and NO_X_ emissions from April–September 2013 averaged tropospheric NO_2_ column data from LOTOS-EUROS (LE) and OMI 2013 observations for different European cities. The MACC-III based emission values, input in LE, are also given.

			OMI			LE		
	Lat	Lon	Lifetime	Top-down E	MACC E	Lifetime	Top-down E	MACC E
	(N)	(E)	(h)	(mg m^−2^ d^−1^)	(mg m^−2^ d^−1^)	(h)	(mg m^−2^ d^−1^)	(mg m^−2^ d^−1^)
Mean			4.1 ± 2.2	5.55 ± 3.49	7.17 ± 3.80	3.7 ± 2.2	7.11 ± 4.03	7.59 ± 3.97
Stdev			1.3 ± 0.7	3.48 ± 2.29	6.29 ± 5.50	1.0 ± 0.9	5.28 ± 3.36	6.09 ± 5.37
Helsinki	60.16	24.93	4.7 ± 2.1	3.88 ± 4.35	4.97 ± 1.62	3.1 ± 1.6	3.78 ± 2.03	4.81 ± 1.70
St-Petersburg	59.93	30.31	3.9 ± 1.5	4.28 ± 1.65	27.02 ± 26.25	3.8 ± 2.2	21.95 ± 12.79	25.57 ± 25.26
Edinburgh	55.94	−3.19	4.2 ± 1.8	6.81 ± 2.92	6.67 ± 2.30	4.8 ± 5.1	5.71 ± 6.16	7.78 ± 2.59
Moscow	55.75	37.61	5.4 ± 2.8	10.91 ± 5.62	11.12 ± 2.67	4.1 ± 3.4	11.21 ± 9.34	11.64 ± 2.21
Amsterdam	52.37	4.89	2.8 ± 1.7	10.41 ± 6.94	15.56 ± 3.88	3.0 ± 2.3	11.04 ± 8.59	15.19 ± 4.25
Warsaw	52.22	22.01	2.9 ± 1.8	3.74 ± 2.28	0.75 ± 0.03	3.7 ± 2.0	0.90 ± 0.48	0.76 ± 0.02
London	51.50	−0.13	4.6 ± 2.5	13.33 ± 7.50	14.97 ± 10.81	3.0 ± 1.7	11.52 ± 6.54	15.08 ± 10.93
Antwerp	51.25	4.38	4.4 ± 2.6	10.22 ± 6.20	12.73 ± 3.19	2.9 ± 2.0	10.85 ± 7.82	12.73 ± 3.19
Brussels	50.83	4.33	4.8 ± 2.3	6.42 ± 3.66	5.09 ± 2.15	3.8 ± 2.5	4.90 ± 3.23	4.98 ± 1.95
Kiev	50.45	30.52	5.4 ± 2.1	3.56 ± 1.63	4.64 ± 0.73	3.9 ± 1.6	2.74 ± 1.13	4.76 ± 0.60
Prague	50.07	14.43	3.3 ± 2.5	3.44 ± 2.60	3.73 ± 1.72	3.7 ± 1.6	2.66 ± 1.17	3.82 ± 1.69
Krakow	50.06	19.94	2.9 ± 1.8	6.56 ± 4.09	4.49 ± 3.15	2.9 ± 1.6	3.12 ± 1.80	5.02 ± 3.49
Paris	48.85	2.35	2.7 ± 1.7	8.80 ± 5.66	7.25 ± 5.98	3.7 ± 2.4	9.98 ± 6.50	7.46 ± 5.06
Vienna	48.20	16.37	7.7 ± 3.3	1.45 ± 0.66	5.66 ± 4.16	5.8 ± 2.5	9.91 ± 5.67	7.32 ± 4.97
Budapest	47.57	19.11	2.9 ± 1.8	3.31 ± 2.25	3.24 ± 0.65	2.6 ± 2.2	2.03 ± 1.72	3.50 ± 0.29
Milan	45.46	9.19	3.8 ± 1.1	/	5.24 ± 0.76	3.9 ± 1.2	8.85 ± 3.67	5.24 ± 0.56
Bucharest	44.43	26.10	3.6 ± 4.0	1.83 ± 2.01	0.59 ± 0.10	2.3 ± 1.9	0.51 ± 0.43	0.59 ± 0.10
Marseille	43.29	5.37	3.4 ± 1.7	/	15.40 ± 6.70	4.1 ± 1.2	14.60 ± 4.31	13.88 ± 6.92
Rome	41.89	12.51	1.8 ± 2.0	7.06 ± 7.73	4.61 ± 1.55	3.5 ± 2.2	11.67 ± 0.35	13.49 ± 7.17
Naples	40.85	14.26	3.1 ± 1.8	3.16 ± 1.85	3.25 ± 2.59	3.4 ± 1.7	4.57 ± 2.34	3.23 ± 2.62
Thessaloniki	40.63	22.94	4.5 ± 1.4	2.19 ± 0.77	2.10 ± 1.02	2.1 ± 1.0	5.15 ± 2.44	2.02 ± 0.93
Madrid	40.41	−3.70	5.7 ± 3.3	4.32 ± 2.48	4.75 ± 5.03	4.6 ± 3.4	4.85 ± 3.62	4.45 ± 4.47
Istanbul	40.00	28.97	4.5 ± 2.2	0.94 ± 0.48	1.12 ± 0.38	6.4 ± 2.3	1.13 ± 0.48	1.18 ± 0.38

No reasonable fit for Milan and Marseille when deriving A from Equation (4).

**Table 2:** Reported lifetimes for different urban areas.

Lifetimes (h)	Location	Source
3.5	Urban areas in the US	[15]
3.8	Urban areas in the US and China	[16]
~3	Madrid	[10]
~4	Moscow	[10]
3.0	Helsinki	[14]
3.0	St-Petersburg	[14]
